# Audiological Performance of ADHEAR Systems in Simulated Conductive Hearing Loss: A Case Series with a Review of the Existing Literature

**DOI:** 10.3390/audiolres11040048

**Published:** 2021-10-13

**Authors:** Enrico Muzzi, Valeria Gambacorta, Ruggero Lapenna, Giulia Pizzamiglio, Sara Ghiselli, Igor Caregnato, Raffaella Marchi, Giampietro Ricci, Eva Orzan

**Affiliations:** 1 Audiology and Otorhinolaryngology Unit, Institute for Maternal and Child Health-Istituto di Ricerca a Carattere Clinico e Scientifico “Burlo Garofolo”, 34137 Trieste, Italy; enrico.muzzi@burlo.trieste.it (E.M.); giuliapizzamiglio93@gmail.com (G.P.); S.Ghiselli@ausl.pc.it (S.G.); raffaella.marchi@burlo.trieste.it (R.M.); eva.orzan@burlo.trieste.it (E.O.); 2 Department of Surgical and Biomedical Sciences, Section of Otorhinolaryngology, University of Perugia, 06129 Perugia, Italy; ruggerolapenna@gmail.com (R.L.); giampietro.ricci@unipg.it (G.R.); 3 Acustica Caregnato, 36063 Marostica, Italy; igor.caregnato@gmail.com

**Keywords:** conductive hearing loss, bone conduction hearing device, otitis media with effusion, binaural hearing

## Abstract

A new non-invasive adhesive bone conduction hearing device (ABCD) has been proposed as an alternative solution for reversible bilateral conductive hearing loss in recurrent or long-lasting forms of otitis media with effusion (OME) in children that cannot undergo surgical treatment. Our aim was to assess the effectiveness of ABCD in children with OME. Twelve normal-hearing Italian-speaking volunteers, in whom a conductive hearing loss was simulated, participated in the study. The free-field average hearing threshold was determined and, to evaluate binaural hearing skills, loudness summation and the squelch effect were assessed. Five conditions were tested: (1) unaided without earplugs, (2) unaided with bilateral earplugs, (3) aided right ear with bilateral earplugs, (4) aided left ear with bilateral earplugs, and (5) bilateral aid with bilateral earplugs. Post-hoc analysis showed a significant statistical difference between plugged, unplugged, and each aided condition. The main results were a better loudness summation and a substantial improvement of the squelch effect in the bilaterally aided. Our results suggest that ABCD is a valid treatment for patients with conductive hearing loss that cannot undergo bone conduction implant surgery. It is also important to consider bilateral aids in order to deal with situations in which binaural hearing is fundamental.

## 1. Introduction

Purely conductive hearing loss is determined by a decrease of the middle ear capacity to transmit sound to the normal inner ear. It can be congenital (e.g., external and/or middle ear malformations), or acquired either during childhood or during the adult life. These last forms are mainly due to inflammatory processes of the middle ear, and they can be permanent (e.g., following ossicular chain erosion due to cholesteatoma) or reversible/fluctuant.

The most representative situation giving a (potentially) reversible bilateral conductive hearing loss is the so-called “otitis media with effusion” (OME). It affects about 90% of children before school age [1], with the highest prevalence rates between 6 months and 4 years of age. Although most episodes of OME resolve spontaneously within 3 months, 30–40% of children experience recurrent events, and in 5 to 10% of cases, last more than one year [2].

When middle ear effusion persists for a long period of time, it can cause a significant decrease in hearing sensitivity, which could result in impaired school performance, failure to respond appropriately to normal conversational speech or environmental sounds, behavioral changes, and possibly a negative impact on the child’s normal speech development [3,4].

Middle ear effusion generally results in a mild conductive hearing loss [5] of approximately 18–35 dB HL [6]. About 50% of patients with a confirmed OME diagnosis present a hearing loss of 20 dB, 20% a hearing loss greater than 35 dB, and 5–10% hearing loss of up to 50 dB [5]. In case of particularly recurrent or long-lasting forms of OME, the insertion of tympanic ventilation tubes (VTs) is considered to be the gold standard treatment VT to significantly improve hearing and reduce the number of OME while in place [2]. However, there are children with specific situations, such as syndromes (e.g., Down S.) or craniofacial disorders, that could have a high anesthesia risk or also a greater recurrence possibility after VT extrusion that does not suggest VT as the best treatment choice. In these patients, hearing aids, and specifically bone conduction devices, represent the alternative solution for the hearing problem. This option gives an excellent audiological benefit but presents disadvantages. 

Recently, a new non-invasive adhesive bone conduction hearing device (ABCD) was proposed to overcome some of the disadvantages of previous disposable bone conduction hearing aids, such as bulkiness and pressure annoyance, with general poor acceptance by the child and the parents [7]. ABCD could potentially be suitable for temporary conductive hearing loss for cases in which surgical treatment cannot be proposed, failed, or should be postponed. Previous studies about ABCD are not very numerous; they deal both with real and simulated conductive hearing loss, both bilateral and unilateral forms, both in the adult and in the children population.

In this paper, we report our experience with the ABCD in a series of subjects with simulated conductive hearing loss with particular reference to binaural listening abilities with unilateral and bilateral aid use. 

## 2. Materials and Methods

### 2.1. Study Subjects and Test Device

Twelve normal-hearing Italian-speaking volunteers, four males and eight females, with a mean age of 31.2 years (range 23–45) participated in the study. In order to simulate a mild-to-moderate bilateral conductive hearing loss, external ear canals were occluded with customized silicone earplugs. Two subjects left the study before completing all the measurements. The ABCD system (ADHEAR, MedEl, Innsbruck, Austria) is a commercially available bone conduction hearing device retained by an advanced adhesive adapter on the hairless skin over the mastoid. The optimal position of the device in the retro auricular area was identified, making sure to avoid contact with the pinna; cleansed and gently rubbed with 70° alcohol; and the adhesive adapter was placed by exerting a slight pressure. The position on the skull should fit the curvature of the adapter well, maximizing the adhesive surface, without any hair underneath

**Figure 1:**
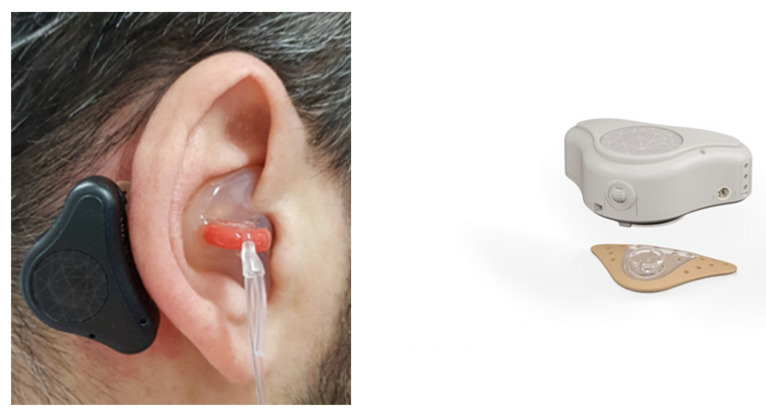
Right ear. ABCD device in place in a typical subject, where the ear canal is occluded by a custom silicone plug. On the right, a commercial figure of the ABCD showing the external laterale surface is visible, and the microphone ports are visible on the right (in the lower part of the device when worn on the right side).

It should be noted that the ABCD is reversible in order to be applied either on the left or on the right but not symmetrical, i.e., the microphone is located in the upper part of the device when applied on the left and in the lower part of the device when placed on the right (Figure 1). 

### 2.2. Hearing Tests

The free-field average hearing threshold at 0.5, 1, 2, and 4 kHz was determined using narrowband noise (NBN) stimuli. 

**Figure 2:**
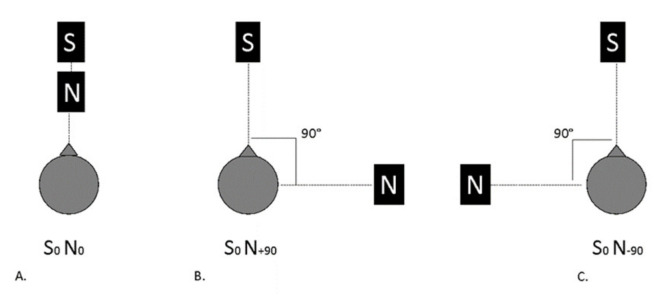
Speech-in-noise test setup. S, signal (speech) source location, N, noise source location. (**A**) Loudness summation test; (**B**) Right squelch effect test; (**C**) Left squelch effect test.

The speech signal consisted of random phonetically balanced lists of 20 spondaic words in the Italian language [8] delivered at 50 dB HL, while pink noise was used as a masker. With the aim of evaluating binaural hearing skills, loudness summation and the squelch effect were tested. To evaluate loudness summation, speech and noise were presented from the same loudspeaker located frontally (Figure 2A). For measuring the squelch effect, both conditions were tested with noise from the right side (+90°, Figure 2B) and from the left side (−90°, Figure 2C), while speech was presented from the front. An adaptive procedure with 2 dB increments/decrements of noise starting from a signal-to-noise ratio (SNR) of 10 dB was employed. The speech reception threshold corresponding to 50% of word recognition (SRT_50_) was taken as the reference target. Overall “best scenario” and “worst scenario” SRT_50_ scores were those obtained in case of unilateral aid use in the conditions of noise lateralized to the unaided side or to the aided side, respectively. The lower the value of SRT_50_, the better the hearing performance in noise. 

Tests were carried out in a soundproof audiometric booth, with loudspeakers positioned at a distance of 1 m from the subject, at the level of the ears. The subjects were advised to avoid head movements during the test. Five conditions were tested: (1) unaided without earplugs, (2) unaided with bilateral earplugs, (3) aided right ear with bilateral earplugs, (4) aided left ear with bilateral earplugs, and (5) bilateral aid with bilateral earplugs.

### 2.3. Statistical Analysis

Results are presented as aided and unaided frequency-specific thresholds expressed as mean values ± SD or median and quartiles (q1; q3) as indicated. The patients’ comparisons were evaluated using two-way ANOVA with post hoc analysis with Tukey test for free field NBN hearing thresholds. Wilcoxon rank sum test (with Bonferroni correction for multiple comparison to avoid type I error) was used to compare SNR50 values with different aided conditions in each tested hearing in noise situation. *p* values < 0.05 were considered significant. Data were analyzed using Excel (version 16.23) and R Commander (version 3.6.0 GUI 1.70) for IOS 10.14.4.

## 3. Results

### 3.1. Free Field Average Hearing Threshold

**Figure 3:**
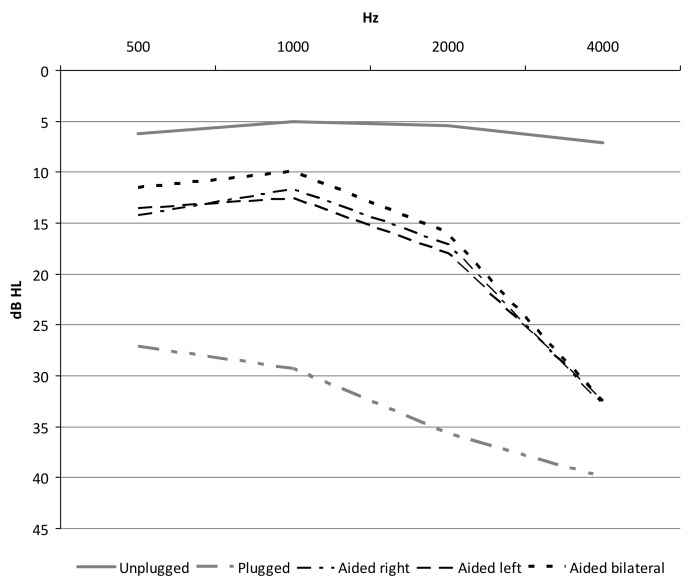
Average free-field threshold in each test condition.

Figure 3 shows the mean free field threshold in each test condition. 

Mean value ± SD values are shown in Table 1.

Given the values of the average NBN audiometry (500, 1000, 2000, 4000 Hz) with plugged ear, post hoc analysis of the power of the sample with respect to the OME population showed a value of 89.1%. 

There was a statistically significant main effect of condition and frequency for the studied audiometry thresholds (two-way ANOVA; *p* < 0.0001). Post-hoc analysis showed a statistically significant difference between the unplugged and plugged conditions, between the plugged and each aided condition, and also between the unplugged and each aided condition (Tukey’s test; *p* < 0.01). No statistically significant differences were found between the three different aided conditions (Tukey’s test; *p* > 0.05). Although there was no difference between the thresholds at each of the frequencies in the unplugged condition (Tukey’s test; *p* > 0.05), there were a statistically significantly higher threshold for 2–4 kHz than 0.5–1 kHz in each study condition (Tukey’s test; *p* < 0.01). However, there was a statistically significant gain for each frequency in the aided conditions (Tukey’s test; *p* < 0.01) 

### 3.2. Binaural Hearing in Noise (Loudness Summation and Squelch Effect)

The median SRT_50_ values (interquartile range) for the loudness summation (S_0_N_0_) and squelch effect (S_0_N_+90_ and S_0_N_–90_) testing are reported in Table 2.

#### 3.2.1. Loudness Summation S_0_N_0_

**Figure 4:**
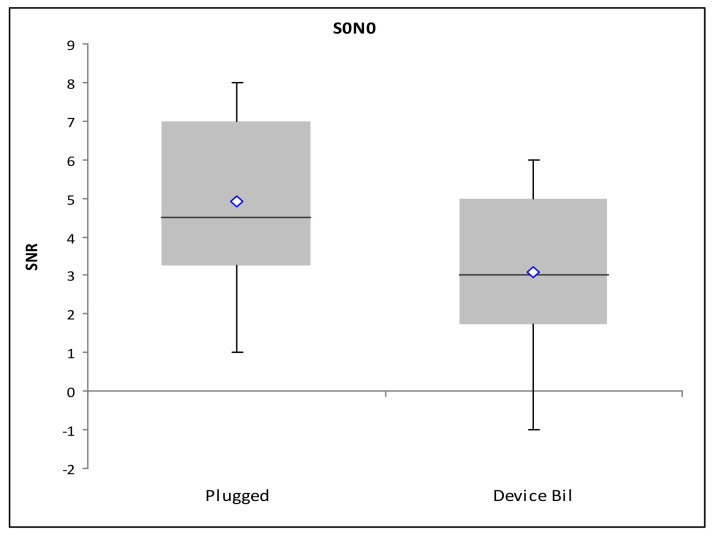
Loudness summation SRT_50_ for the unaided and bilaterally aided plugged condition.

There is a statistically significant difference for the SRT_50_ values between the unplugged and plugged conditions (*p* < 0.0125; Wilcoxon rank sum test with Bonferroni correction). The greatest improvement is reached with the bilaterally aided condition, even if a clear statistical significance is not reached (*p* = 0.051; Figure 4). However, SRT_50_ with the bilateral aid does not statistically differ from the unplugged condition, while SRT_50_ is statistically significantly higher for both unilateral aided conditions with respect to the unplugged one (*p* < 0.0125).

#### 3.2.2. Squelch Effect (S_0_N_+90_)

There is a statistically significant difference for the SRT_50_ values between the unplugged and plugged conditions (*p* < 0.0125; Wilcoxon rank sum test with Bonferroni correction). A statistically significant improvement compared to the plugged condition is reached only in the bilaterally aided condition (*p* < 0.0125; Wilcoxon rank sum test with Bonferroni correction). SRT_50_ with the bilateral aid and with the aid on the left (best scenario) is not statistically different from the unplugged condition (*p* >0.0125). SRT_50_ is statistically significantly higher for the right aided condition (worst scenario; *p* < 0.0125) compared both to the unplugged condition and to the bilaterally aided one (*p* < 0.0125; Wilcoxon rank sum test with Bonferroni correction).

#### 3.2.3. Squelch Effect (S_0_N_−90_)

There is a statistically significant difference for the SRT_50_ values between the unplugged and plugged conditions (*p* < 0.0125; Wilcoxon rank sum test with Bonferroni correction). The greatest improvement is reached with the bilaterally aided condition, even if a clear statistical significance is not reached (*p* = 0.09). SRT_50_ with the bilateral aid and with the aid on the right (best scenario) is not statistically different from the unplugged condition (*p* > 0.0125). SNR_50_ is statistically significantly higher for the left aided condition (worst scenario; *p* < 0.0125) compared both to the unplugged condition and to the bilaterally aided one (*p* < 0.0125; Wilcoxon rank sum test with Bonferroni correction).

**Figure 5:**
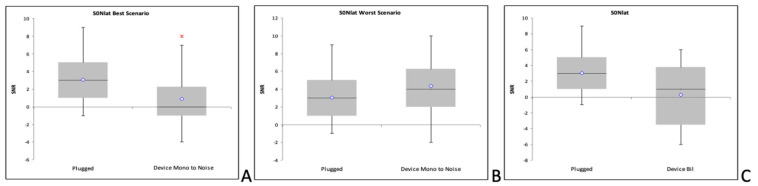
Aided squelch SRT50. (**A**) Overall scores with noise lateralized to the unaided ear, or “best scenario”; (**B**) Overall scores with noise lateralized to the aided ear, or “worst scenario”; (**C**) Overall bilaterally aided condition.

Figure 5 graphically shows the overall SRT_50_ scores in the aided conditions in the different situations of noise localization. It can be seen that there is a worsening of SRT50 in the worst noise source scenario compared to the plugged condition.

## 4. Discussion

The benefit derived from ABCD when compared to the simulated unaided conductive hearing loss measurements is clear.

In the present paper, we simulated bilateral conductive hearing loss in normal hearing subjects to assess the effectiveness of ABCD, for example, in children with OME, which potentially leads to a poorer quality of life in the patient and could negatively influence daily life, especially when binaural hearing is necessary (e.g., school).

Air conduction hearing devices have been traditionally successfully adopted in cases of CHL due to chronic or particularly recurrent forms of OME. However, bone conduction hearing aids overcome some disadvantages of external ear canal occlusion, allowing the necessity of regulation of the gain in relation to the possible fluctuation of the air-bone gap to be avoided. In the presence of normal bone conduction, BC hearing aids do not necessitate any regulation due to the different aid conduction threshold at any time.

The study was conducted in young adults because of the number of tests conducted that would not be tolerated by children. The conclusions are supposed to be relevant for children with bilateral conductive hearing loss as well because even if young children need a more favorable signal-to-noise ratio than adults [9], the OME-related hearing loss simulation method in children leads to a comparable speech perception impairment [10].

Other studies have demonstrated that speech perception performance in children with simulated hearing impairment is similar to that of children with OME [6]. Therefore, the simulated conductive hearing loss can be used to compare the hearing abilities and potential speech perception impairment of children with OME. We successfully simulated a mild to moderate hearing loss (average 33.75 dB HL), in a range similar to what is expected in case of OME (18–35 dB HL) using an earplug. Then, we tested hearing abilities in noise and in quiet environments. 

With reference to the free field NBN hearing thresholds, a significant difference between the unplugged and plugged conditions and between plugged and each of the aided conditions was observed. Instead, there was no difference in the hearing threshold between the aided conditions. There was also a difference in the gain obtained with the use of ABCD for the individual frequencies. A better gain was observed in the low and mid frequencies compared to 4 kHz. Previous studies about ABCD application in CHL [11,12] obtained similar results. This means that normal hearing is still better than the aided one and that limitations of transcutaneous bone conduction are still present. 

Most previous studies about ABCD application were heterogeneous regarding the included subjects (age and hearing loss etiology), ABCD side application procedure, and hearing abilities tested.

The main novelty of this study was testing the binaural hearing abilities in different experimental situations, including the bilaterally aided condition and different combinations of noise sources when testing the hearing in noise ability. The main results were a better loudness summation and a substantial improvement of the squelch effect in the bilaterally aided condition compared to the other conditions tested. 

Binaural hearing is based on spatial auditory cues, such as interaural time difference and interaural level difference between the two ears, that can help a human localize the sound source. In case of binaural impairment, skills, such as recognizing speech in noise or localizing the direction of sound, become more difficult. For this reason, Snapp et al. [13] stated that bilateral hearing stimulation is considered the gold standard to achieve excellent auditory performance.

Bilateral bone conduction hearing aid application (e.g., ABCD) in case of bilateral conductive hearing loss allows binaural hearing to be restored and overcomes such environmental noise situations in which a unilateral hearing aid would be paradoxically disadvantageous (e.g., the noise source on the side of the hearing aid). In this sense, our study confirms Neumann et al.’s findings [14]. 

Other studies about ABCD [12,15,16,17] also compared them to conventional bone conduction hearing aids (BCHAs) in subjects with true or simulated conductive hearing loss. They found that audiologic assessment of aided sound field thresholds, SRTs in quiet and in noise, and WRSs showed no statistically significant differences when comparing the two devices. In particular, the ABCD also showed better results than the BCHA on a soft-band and therefore seems to be a promising solution for children with CHL aged below 10 years [14]. Comparing ABCD and BCHA, a statistically significant difference concerning daily usage was also found: the median reported wearing time of the adhesive device was 8.1 h compared to 4.3 h of conventional BCHA usage [15]. 

ABCD is a safe and effective device to treat conductive hearing loss and may considerably improve the quality of life for patients affected by OME. This device is well tolerated, its pressure-free nature could be an advantage over the other BCHA, causing no pain or skin irritation for the majority of patients. 

However, the literature shows some limits of this device. Dahm et al. estimated an average battery durability of 5.9 days [11], but as stated by Neumann et al., excessive handling of the adapter, mastoid shape, skin type, and sweating could cause variation [14]. As stated by Mertens et al., for optimal retention of the adhesive adapter, special attention should be paid to the skin preparation (clean and dry) and correct placement [18].

For scientific and consultation purposes, a comprehensive review of the existing literature about ABCD is reported in Table 3.

## 5. Conclusions

Our results suggest that ABCD is a valid treatment for patients with conductive hearing loss that cannot undergo bone conduction aid implant surgery. It is also important to consider bilateral aids in order to deal with situations in which binaural hearing is fundamental.

## Figures and Tables

**Table 1:** Free Field NBN audiometry results for tested frequencies in each condition.

	500 Hz	1000 Hz	2000 Hz	4000 Hz
Unplugged	6.25 ± 2.26	5 ± 3	5.42 ± 3.96	7.08 ± 3.34
Plugged	28.3 ± 6.85	27.14 ± 7.48	36.25 ± 6.9	42.5 ± 6.9
Aided right	14.17 ± 2.8	11.67 ± 3.25	17.08 ± 3.96	32.92 ± 4.98
Aided left	13.5 ± 2.4	12.5 ± 3.5	18 ± 3.5	32.5 ± 5.9
Aided bil	11.5 ± 3.37	10 ± 2.35	16 ± 5.6	32.5 ± 4.24

**Table 2:** Median SNR_50_ values (interquartile range) at loudness summation (S_0_N_0_) and squelch effect (S_0_N_+90_ and S_0_N_−90_) testing.

	Loudness Summation S_0_N_0_	Squelch Effect (S_0_N_+90_)	Squelch Effect (S_0_N_–90_)
Unplugged	2 dB (0; 2.3)	–5 dB (–7; –2)	–2 dB (–3.5; –1)
Plugged	4.5 dB (3.8; 7)	3 dB (2; 5)	3 dB (0.75; 5)
Aided right	3 dB (3; 6.3)	4 dB (3; 6)	0 dB (–1; 1.5)
Aided left	5.5 dB (4; 7.8)	0 dB (0; 2),	5 dB (2.25; 5.75)
Aided bil	3 dB (2.3; 4.8)	0 dB (–4; 3)	1 dB (–1.5; 3.5)

**Table 3:** Comprehensive review of the existing literature about ABCD.

Authors	Subjects	Aided Condition Tested	Tests	Results
Brill et al. 2019 [19]	N = 12 Age = Adult Simulated with bilateral conductive hearing loss with a foam earplug	Unilateral ABCD.	- Free field tone audiometry - Number perception - Monosyllable perception	- Improvement in free-field hearing thresholds and significant tone audiometry gain.
Weiss et al. 2019 [20]	N = 11 Age = 18 years of age or older. Transient conductive hearing loss due to auditory canal tamponade after middle ear surgery.	Unilateral ABCD at the tamponade side, with contralateral ear plugged and covered.	- Free field tone audiometry. - Speech reception thresholds (SRT) in quiet and SRT in noise S_0_N_0._ - Speech, Spatial, and Qualities of Hearing 12 questionnaire.	- Speech perception for monosyllables in quiet improved. - Functional hearing gain improved. - Speech perception in noise improved. - The results of the questionnaire showed a high level of patient satisfaction and subjective hearing improvement.
Almuhawas et al. 2020 [21]	N = 12 Age = between 5 and 53 years. Conductive hearing loss (different etiologies).	Unilateral ABCD with the contralateral ear occluded with specific earplugs.	- Free field tone audiometry. - Speech reception threshold in quiet and noise. - Speech, Spatial, and Qualities of Hearing 12 questionnaire (SSQ12).	- Overall improvement in the aided thresholds when compared to the unaided hearing thresholds in the sound field. - Significant difference in speech perception in free field - Significantly higher word recognition scores in the aided condition - The results of the patient surveys using SSQ12 questionnaires demonstrated improved auditory performance hearing sensation and a high satisfaction rate for the system
Dahm et al. 2018 [11]	N = 12 Age = between 14 to 74 years. Bilateral or unilateral conductive hearing loss (different etiologies).	Unilateral ABCD, with the contralateral ear covered with a circumaural earmuff or with the application of a masking signal.	- Free field tone audiometry - Speech reception threshold (SRT) in quiet and in noise - Speech, Spatial, and Qualities of Hearing 12 questionnaire.	- Hearing gain at free field audiometry and SRT. - The sound field comparison to a conventional softband BCHA showed comparable levels of benefit.
Dahm et al. 2019 [15]	N = 13 Age = between 12 to 63 years Unilateral or bilateral conductive hearing loss	Unilateral ABCD, with the application in the contralateral ear of a masking signal Unilateral BCHA, with the application in the contralateral ear of a masking signal.	- Free field tone audiometry. - Speech reception threshold in quiet and in noise. - Speech, Spatial, and Qualities of Hearing 12 questionnaire. - Assessment of Quality of Life-8 Dimensions questionnaire.	- Statistically significant difference concerning the daily usage between an ABCD and a BCHA. - No statistically significant audiological difference between the two devices.
Favoreel et al. 2020 [16]	N = 10 Age = between 4 to 17 years. Unilateral or bilateral conductive hearing loss.	Unilateral ABCD with contralateral ear closed with an earplug and headphones. Unilateral BCHA with contralateral ear closed with an earplug and headphones.	- Free field tone audiometry. - Speech audiometry in quiet. - - Speech, Spatial, and Qualities of Hearing 12 questionnaire.	- Hearing improvements with the ABCD and the BCHA on a softband. - No significant difference between the ABCD and the BCHA.
Kuthubutheen et al. 2020 [12]	N = 12 Age = between 11 to 70 years. Unilateral conductive hearing loss.	Unilateral ABCD. Unilateral BCHA.	- Free field tone audiometry. - Speech audiometry in quiet and in noise (S_0_N_0_). - Speech, Spatial, and Qualities of Hearing 12 questionnaire.	- Significant improvements in pure-tone thresholds as well as speech understanding both in quiet and in noise with both devices.
Neumann et al. 2019 [14]	N = 10 Age = between 3 months to 10 years. Unilateral or bilateral conductive hearing loss.	Unilateral ABCD, with the application in the contralateral ear of a masking signal. Unilateral BCHA with the application in the contralateral ear of a masking signal.	- Free field tone audiometry. - Speech audiometry in quiet and in noise. - LittlEARS Auditory Questionnaire. - Speech, Spatial and Qualities of Hearing Scale Questionnaire for parents.	- Functional gain with the ABCD exceeded that of the BCHA. - Speech perception in quiet and noise improved in the aided situation similarly for both hearing devices.

## Data Availability

The data presented in this study are available on request from the corresponding author.
